# Compression de la moelle épinière secondaire à une métastase d’un lymphome de Burkitt gingival révélant un cas d’infection par le VIH au Maroc

**DOI:** 10.48327/mtsi.v2i1.2022.203

**Published:** 2022-02-04

**Authors:** Imane SELLAM, Soufiane ABDOUH, Ibrahim ABOUHALI, Mohamed AKSIM, Mouna ELFANE

**Affiliations:** Service des maladies infectieuses, Faculté de médecine et de pharmacie, Université Ibn Zohr, Agadir, Maroc

**Keywords:** Lymphome de Burkitt, Infection à VIH, Chimiothérapie, Traitement antirétroviral, Maroc, Maghreb, Afrique du Nord, Burkitt lymphoma, HIV infection, Chemotherapy, Antiretroviral treatment, Morocco, Maghreb, Northern Africa

## Abstract

Les localisations buccales du lymphome de Burkitt sont rarement observées et rapportées dans la littérature chez les patients infectés par le VIH. Le tableau clinique peut évoquer d’autres tumeurs ou infections gingivales, ce qui retarde le diagnostic et la prise en charge. L’atteinte de la moelle épinière est rare, et seulement quelques cas sont rapportés dans la littérature. Nous rapportons un cas de lymphome de Burkitt gingival avec atteinte de la moelle épinière révélant l’infection à VIH chez une patiente âgée de 44 ans et qui a bien évolué sous chimiothérapie et traitement antirétroviral.

## Abstract (en)

Burkitt lymphoma in HIV-infected patients is seldom seen in the oral cavity, and they are rarely reported in the literature. The clinical manifestations may suggest other tumors or gingival infections, which delays diagnosis and management. Spinal cord involvement is exceptional and only a few cases are reported. We report a case of Burkitt lymphoma in the oral cavity and spinal cord, revealing HIV infection in a 44-year-old patient, who responded well to chemotherapy and antiretroviral treatment and is on remission.

## Introduction

Le lymphome de Burkitt (LB) est un lymphome appartenant au groupe des lymphomes malins non hodgkiniens (LMNH) à cellules B. Les localisations maxillo-faciales et abdominales sont les plus fréquentes même si l’affection peut intéresser tous les organes et des études signalent la rareté des localisations neuro-méningées dont l’atteinte de la moelle épinière [4, 7, 10]. Nous rapportons un cas de lymphome de Burkitt révélé par une compression de la moelle épinière, associée à une masse tumorale maxillo-faciale, révélant une infection à VIH.

## Observation

**Figure 1:**
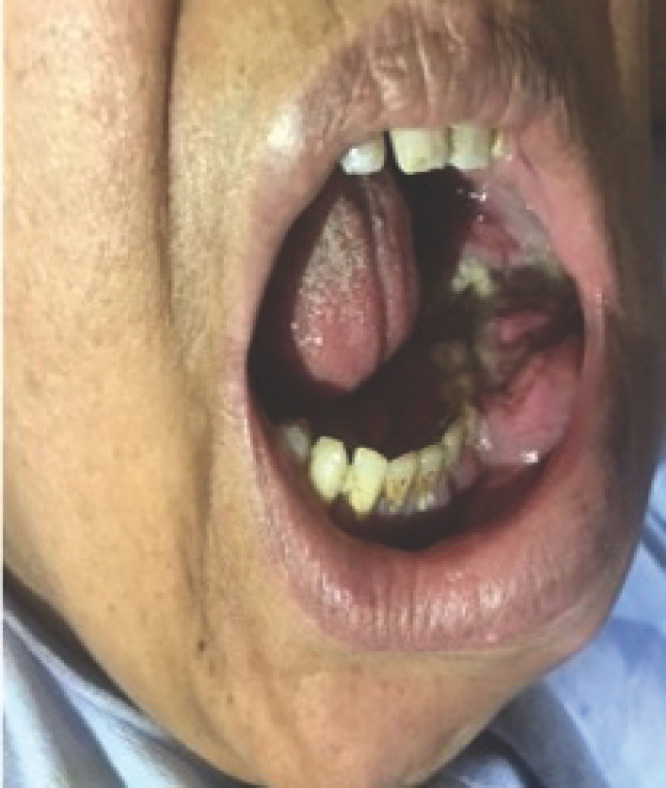
Aspect macroscopique de la masse tumorale maxillo-faciale gauche Macroscopic appearance of the maxillofacial tumor mass

**Figure 2:**
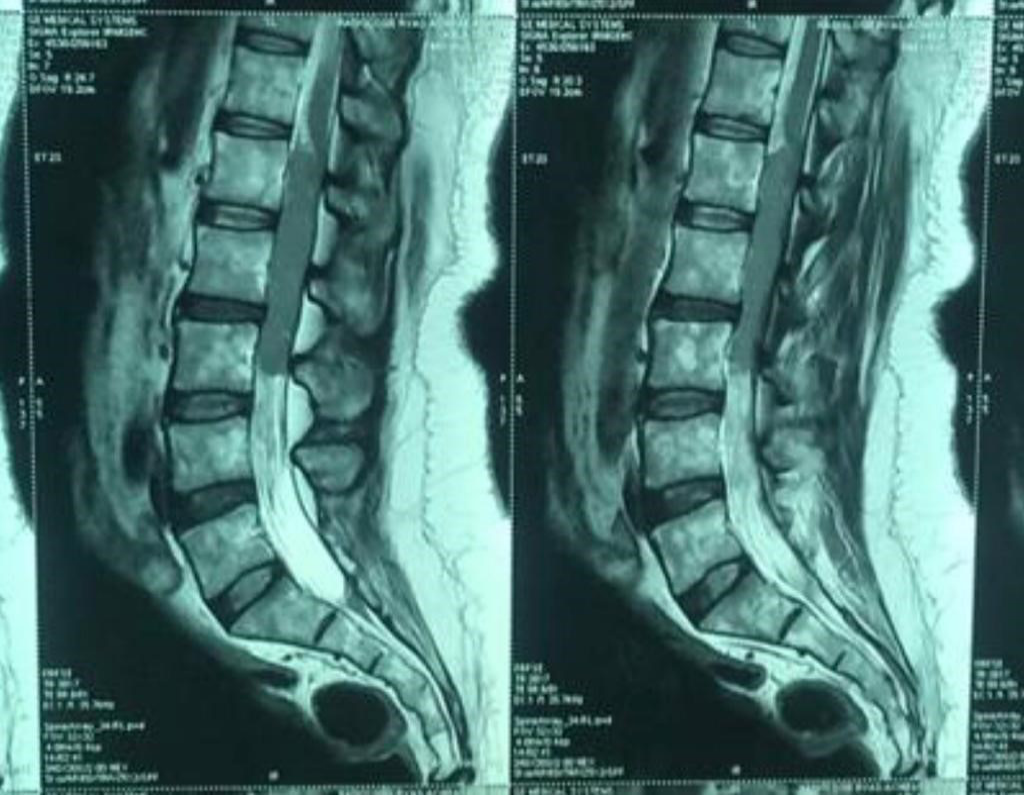
Imagerie par résonance magnétique médullaire en séquence T2 montrant un processus lésionnel intradural et extra médullaire développé en avant et en dessous du cône médullaire MRI, magnetic resonance imaging in T2 sequence showing an intradural and extra medullary lesion process developed in front of and below the medullary cone

**Figure 3:**
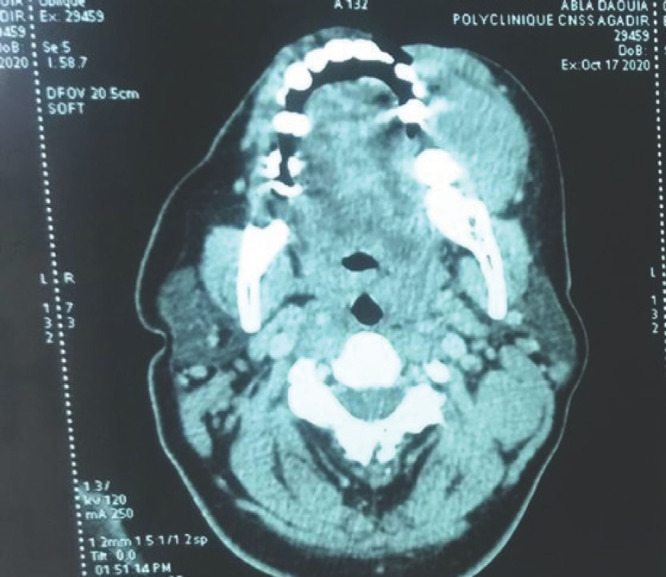
Tomodensitométrie cérébro-faciale montrant un processus tumoral gingivale gauche au contact du corps mandibulaire Cerebrofacial computed tomography showing a left gingival tumor process in contact with the mandibular body

**Figure 4:**
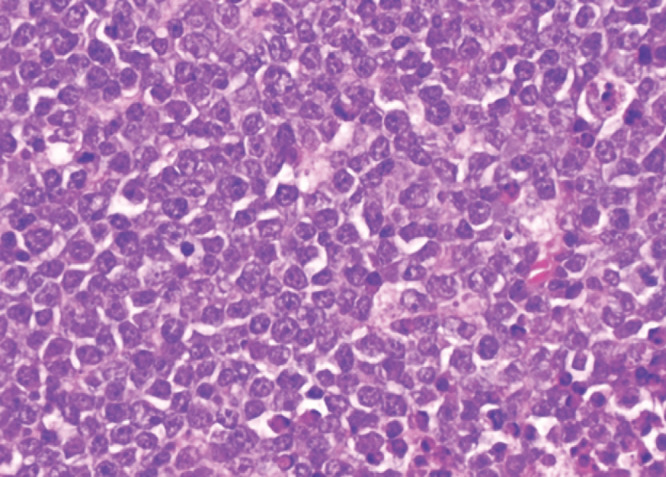
H.E x 20: H.E x 40: cellules de taille moyenne pourvues de nombreuses mitoses H&E x 40: medium-sized cells with numerous mitoses

**Figure 5:**
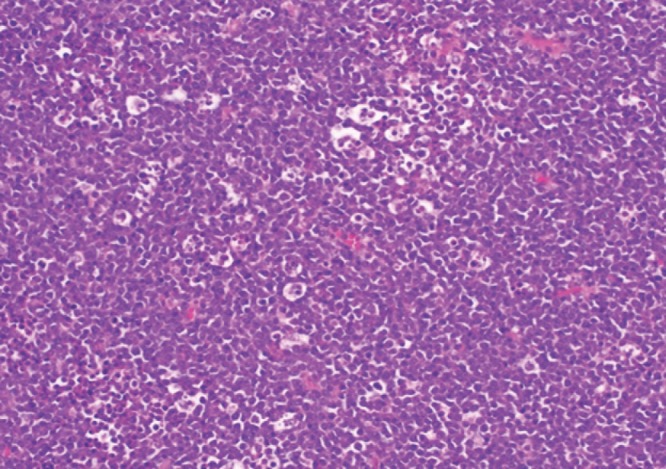
H.E x 20: prolifération lymphomateuse diffuse et dense avec aspect en ciel étoilé H&E x 20: diffuse and dense lymphomatous proliferation with a starry sky appearance

Notre observation concerne Madame A. D., femme au foyer, âgée de 44 ans, divorcée et mère de deux enfants, ayant des rapports hétérosexuels multiples non protégés. La patiente était admise pour une paraplégie flasque. Le début des symptômes remontait à cinq mois avant son admission par l’apparition d’un gonflement de la gencive mandibulaire avec un inconfort local et saignement suite à des extractions dentaires. Par ailleurs la patiente avait présenté trois mois avant son admission des douleurs à type de paresthésies intenses, d’installation progressive, dorsolombaires, irradiant aux fesses, à la face postérieure des cuisses et des jambes, avec installation progressive d’une impotence fonctionnelle des deux membres inférieurs évoluant vers une paraplégie. À l’admission, l’examen neurologique retrouvait une patiente consciente avec une paraplégie, des réflexes ostéotendineux vifs, une sensibilité tactile épicritique et thermo-algique normale. L’intensité de la douleur était évaluée à l’aide de l’échelle EVA, la patiente avait un score égal à 10, ce qui exprimait une intensité douloureuse subjective insupportable. L’examen des paires crâniennes notait un strabisme convergent. L’examen de la cavité buccale retrouvait une masse gingivale inférieure gauche bourgeonnante fixe et mesurant 4x3 cm, avec un saignement au contact (Fig. 1). On notait également un déchaussement dentaire et la présence d’une adénopathie jugulo-carotidienne homolatérale à la masse gingivale, mesurant 2x2 cm, de consistance solide et mobile par rapport au plan profond et superficiel. L’imagerie par résonance magnétique (IRM) de la moelle épinière avait montré un processus lésionnel intradural et extra-médullaire développé en avant et en dessous du cône médullaire réalisant une masse tissulaire hétérogène étendue. Ce processus était en iso-signal T1 et discret hypersignal T2 (Fig. 2) et STIR rehaussé de façon modérée après injection de Gadolinium. L’IRM cérébrale avait montré un conflit neuro-vasculaire entre le nerf abducens (VI) gauche et l’artère basilaire. La ponction lombaire avait montré une méningite lymphocytaire (23 éléments/mm^3^) avec hyperprotéinorachie à 2,58 g/l et une hypoglycorachie (rapport glycorachie/glycémie < 40 %), la culture était négative. La tomodensitométrie cranio-faciale avait montré un processus lésionnel gingival gauche au contact du corps mandibulaire avec adénopathie homolatérale (Fig. 3). La biopsie de la masse maxillo-faciale suivie d’un examen anatomopathologique retrouvait une muqueuse malpighienne infiltrée par une prolifération lymphomateuse avec des cellules de taille moyenne pourvues de cytoplasmes basophiles et de noyaux hyperchromatiques, aux contours irréguliers avec de très nombreuses mitoses et caryorrhexie (Fig. 4). Il s’y associait des macrophages à corps tangibles réalisant un aspect en ciel étoilé (Fig. 5). À l’immunohistochimie, la prolifération tumorale lymphoïde était de phénotype B CD79a, CD20 positive, réalisant un marquage diffus en réseau. Cette prolifération exprimait CD10 de façon diffuse en réseau et BCL6 de manière diffuse. Elle avait montré un index de prolifération élevé. Dans le cadre du bilan d’extension du lymphome, l’hémogramme était normal.

Le scanner thoraco-abdomino-pelvien avait montré un épaississement gastrique et intestinal avec une masse surrénalienne et ovarienne. La biopsie ostéomédullaire (BOM) avait montré une hypoplasie médullaire sans malignité. Le bilan de lyse tumorale avait montré une hypokaliémie 3,39 mmol/l, phosphorémie normale à 39,7 mg/l, calcémie normale à 8,53 mg/dl, urée normale à 0,16 g/l, créatininémie normale à 0,6 mg/dl et LDH (lactate déshydrogénase) élevée à 902 U/l. Le bilan pré-thérapeutique était sans particularité, l’électrocardiogramme et l’échodoppler cardiaque étaient normaux de même que la fonction rénale. Dans le cadre du bilan initial de l’infection à VIH, la sérologie VIH (ELISA et Western Blot) était positive avec une charge virale initiale VIH à 133 913 copies/ml et un taux des lymphocytes TCD4 à 400 cellules/mm^3^. Le bilan de la tuberculose (recherche de bacille de Koch dans les expectorations, radiographie du thorax) était négatif. Les sérologies des hépatites B, C, de la syphilis étaient négatives. L’examen du fond d’œil était normal. Une trithérapie antirétrovirale était initiée en premier lieu par l’association éfavirenz/emtricitabine/ténofovir puis, 2 mois plus tard, une polychimiothérapie à base de cyclophosphamide, hydroxyadriamycine, vincristine et prednisone (CHOP) était débutée. La patiente avait reçu au total 6 cures du protocole CHOP. L’évolution clinique après 18 mois était favorable, marquée par une régression de la masse gingivale et une récupération de l’impotence fonctionnelle.

## Discussion

Le cas ci-dessus décrit un syndrome de compression de la moelle épinière, avec paraplégie survenue dans un contexte de masse tumorale maxillo-faciale chez une jeune femme de 44 ans. Le diagnostic de lymphome de Burkitt stade IV a été retenu. Le lymphome de Burkitt (LB) est un lymphome non hodgkinien (LNH) de haut grade de malignité. On distingue trois formes du lymphome de Burkitt: endémique, sporadique et chez l’immunodéprimé. L’atteinte ganglionnaire et neuroméningée est plus fréquente dans le lymphome de Burkitt associé au VIH que dans le lymphome de Burkitt endémique, qui est plus fréquent chez les patients séronégatifs [14]. La première manifestation du lymphome de Burkitt associé au VIH est souvent liée aux sites extranodaux [1, 11]. Les sites extranodaux les plus courants sont le tractus gastro-intestinal et la moelle osseuse [1]. Les sites intraoraux privilégiés sont les muqueuses palatales et l’os. La gencive est l’un des sites intraoraux les plus rares – cela s’explique en partie par le fait que le tissu lymphoïde n’est normalement pas retrouvé dans la gencive [2, 15]. Le risque de développer un lymphome de Burkitt chez les patients séropositifs est en effet 50 fois supérieur à celui de la population générale. Les principaux facteurs favorisant la survenue du LNH au cours de l’infection à VIH sont le dysfonctionnement immunitaire, les virus oncogènes tels que le virus Epstein Barr (EBV), l’herpès virus humain type 8 (HHV8) et les anomalies moléculaires et cytogénétiques secondaires à l’infection rétrovirale [1, 11]. Le VIH peut induire une activation chronique des cellules B responsable d’un dysfonctionnement immunitaire qui conduit à une expansion clonale dysrégulée des lymphocytes B. La stimulation persistante incontrôlée des lymphocytes B peut favoriser la prolifération de cellules B monoclonales [11]. Le risque de développement du lymphome est lié à la réplication virale, indépendamment du taux de CD4 [6,7,9,15]. Les atteintes neuro-méningées dans le lymphome de Burkitt représentent souvent une localisation secondaire et définissent un stade évolué de la maladie [3,4,5,13]. Dans notre cas, le bilan d’extension clinique a retrouvé une adénopathie jugulo-carotidienne et le bilan paraclinique a montré un épaississement gastrique et intestinal et une masse surrénalienne et ovarienne. Les cas révélés par une compression de la moelle épinière ont surtout été rapportés chez l’enfant dont les 7 cas rapportés par Ses et al, où la moyenne d’âge était de 15 ans [5]. Plusieurs hypothèses ont été avancées. Pour certains auteurs, il s’agissait d’une transformation lymphomatose d’un tissu lymphoïde préexistant dans l’espace épidural. Pour d’autres, les masses épidurales résulteraient d’une extension tumorale à partir de localisations osseuses et paravertébrales [4]. Dans notre cas, il s’agit en effet d’un LNH gingival de type Burkitt, stade IV avec extension épidurale et compression du cône médullaire sans atteinte vertébrale. Le diagnostic doit être fait en urgence par l’histologie et l’immunohistochimie. En anatomopathologie, le lymphome de Burkitt est d’identification presque exclusivement morphologique: cellules de taille moyenne, avec un noyau régulier, une chromatine réticulée immature comportant quelques nucléoles en situation souvent centrale. Il existe une importante basophilie du cytoplasme avec un aspect typique en « ciel étoilé » provoqué par la clarté des macrophages réactionnels dispersés dans une population tumorale dense et basophile [4, 7]. Les études immunohistochimiques ont permis de démontrer que les cellules du lymphome de Burkitt exprimaient les antigènes de différenciation des cellules de la lignée B: CDI9, CD20, CD22, CD79a ainsi que CDI0, en l’absence d’expression des antigènes de différenciation de la lignée T [7,8,12,14]. Le caryotype de notre patiente montre que la prolifération tumorale exprime CD10 + et BCL6 sans expression de l’ADN polymérase, la désoxynucléotidyl-transférase terminale (TDT). La prise en charge thérapeutique de la compression médullaire résultant du lymphome de Burkitt peut faire appel à la laminectomie décompressive. Pour Dechambenoit [4], la chirurgie ne se justifie qu’à titre diagnostic. Dans notre cas, le diagnostic a été fait par la biopsie de la masse gingivale [4]. L’association radiothérapie-chimiothérapie a prouvé son efficacité sur l’amélioration de la survie globale des patients [6,7,9,12,16]. Nous n’avons pas eu recours à la radiothérapie du fait de la bonne évolution sous chimiothérapie seule. Le traitement antirétroviral est indispensable à la prise en charge des LMNH [7, 9] car une réplication virale persistante est un facteur de mauvais pronostic [15]. Le pronostic des patients atteints de lymphomes B à grandes cellules traités par chimiothérapie et un traitement antirétroviral hautement actif [HAART] est aujourd’hui similaire à celui des patients non infectés par le VIH recevant une même chimiothérapie [6]. Cependant dans la série de Shah, l’infection par le VIH a été un facteur de mauvais pronostic. Les deux patients infectés par le VIH sont décédés [15]. L’évolution après 18 mois de notre patiente était bonne.

## Conclusion

Ce cas clinique montre que le LNH est devenu un mode de plus en plus fréquent de la découverte de l’infection à VIH, d’où la nécessité de rechercher systématiquement l’infection à VIH devant tout cas de lymphome. La prise en charge précoce par un traitement antirétroviral et une chimiothérapie permet d’améliorer l’espérance de vie des patients infectés par le VIH et atteints de LNH.

## Liens d’intérêts

Les auteurs ne déclarent aucun lien d’intérêt.

## Contribution des auteurs

Imane SELLAM: Rédaction, suivi clinique, prospection bibliographique. Mohamed AKSIM: exécution des tests de laboratoire Soufiane ABDOUH: prospection bibliographique. Ibrahim ABOUHALI: co-rédaction. Mouna ELFANE: supervision, co-rédaction, relecture et validation du manuscrit, suivi clinique
